# Only strict quarantine measures can curb the coronavirus disease (COVID-19) outbreak in Italy, 2020

**DOI:** 10.2807/1560-7917.ES.2020.25.13.2000280

**Published:** 2020-04-02

**Authors:** Henrik Sjödin, Annelies Wilder-Smith, Sarah Osman, Zia Farooq, Joacim Rocklöv

**Affiliations:** 1 Department of Public Health and Clinical Medicine, Umeå University, Umeå, Sweden; 2 Department of Epidemiology and Global Health, Umeå University, Umeå, Sweden; 3 Department of Disease Control, London School of Hygiene and Tropical Medicine, United Kingdom; 4 Heidelberg Institute of Global Health, University of Heidelberg, Germany

**Keywords:** coronavirus, SARS-CoV-2, COVID-19, Outbreak, isolation and quarantine, Social distancing

## Abstract

Several Italian towns are under lockdown to contain the COVID-19 outbreak. The level of transmission reduction required for physical distancing interventions to mitigate the epidemic is a crucial question. We show that very high adherence to community quarantine (total stay-home policy) and a small household size is necessary for curbing the outbreak in a locked-down town. The larger the household size and amount of time in the public, the longer the lockdown period needed.

## European epicentre

In February 2020, Italy became the epicentre for coronavirus disease (COVID-19) in Europe, with many exportations to other countries, and widespread community transmission, particularly in Northern Italy [1]. As a public health response, on 22 February 2020, Italy imposed a lockdown with shutdown of businesses, schools and public places plus physical distancing in ‘hotspot’ regions close to Milan and Venice. Approximately 50,000 people could not enter or leave several towns in Veneto and Lombardy for 14 days without special permission. The population sizes in these towns range from 927 to 15,293 individuals [2]. As at 9 March 2020, 7,375 laboratory-confirmed cases of COVID-19 and 366 deaths had been observed in Italy, so on that date, the community quarantine was extended to include all of Northern Italy until 3 April. Here we aim to investigate the extent of physical distancing needed to effectively control the outbreak in a lockdown situation in a small size town setting typical of Italy. We specifically estimate the disease burden and the time required until the quarantine can be lifted, by taking into account the time spent by individuals in the public (i.e. outside of the home) and the household size.

## Stochastic individual based processes

To account for the importance of stochasticity in individual-based processes within smaller cities and within households, we modelled the outbreak progression as a continuous-time Markov process, specifically by developing a susceptible-exposed-infectious-recovered (SEIR) epidemiological model in the form of a Master equation [3-5] (Supplement 1). The model was parameterised to COVID-19 based on published data on incubation time and infectious period [6,7]. We modelled a scenario where 0.1% of the population in the quarantined town would be in the latent period (i.e. the period of time between the point of infection and the onset of infectiousness) at the time of implementing the quarantine policy on 22 February, and that all symptomatic cases would have been moved out the locked-down town (e.g. placed in a hospital for care and isolation). This corresponds to five latent persons in a city of 5,000 persons. Further, we assumed that all persons were isolated after 1 day of symptoms. We also assumed a pre-symptomatic period of infectiousness of 1 day. Of all infected persons, we modelled different proportions of asymptomatic infections (scenarios of 10%, 20% or 50%) based on our preliminary knowledge on such proportions [8,9]. Persons with asymptomatic infections would not be isolated and continue to contribute to transmission. Parameters are summarised in the Table.

**Table:** Most central parameters and corresponding values used for the model of COVID-19 outbreak progression in this study

Parameter	Value
Incubation period in days	5
Latent period (𝐿) in days	4
Infectious period (1/𝛾) in days	2
Infectious period of asymptomatic persons (1/𝛾_𝑎_) in days	10
Proportion asymptomatic (𝑎)	(0.1, 0.2, 0.5)
Within household contact-rate (𝑐)	2.1⁄𝜏
Public location contact-rate (𝑐̃)	0.27⁄𝜏

COVID-19: coronavirus disease.

The parameter γ denotes the daily rate of recovery from the disease, 𝛾_𝑎_ denotes the daily rate of removal to e.g., a hospital, and τ denotes the probability of virus transmission from an infectious person to a susceptible in the event of contact (See Supplement 1).

Importantly, the standard reproductive rate [5], equal to the product between contact rate and the probability 𝜏 of transmission given a contact event, was set within a household to β = cτ = 2.1 [10], and within public locations in the community to $\tilde{\beta }=\tilde{c}\tau =0.27$; lower than in mainland China or on the cruise ship Diamond Princess [10,11], as population densities in European towns are lower. The within-household contact-rate, 𝑐, was thus assumed to be eight times higher than the contact-rate 𝑐̃ at public locations. We could then apply a quarantine adherence parameter 𝜑, to model dynamically the amount of time spent in households relative to that in public locations (see Supplement 1 for a more detailed description).

## Quarantine scenarios

We modelled the effectiveness of quarantine based on the degree of adherence to quarantine, measured by the number of hours per person spent in the public per day. Complete noncompliance to community quarantine corresponds to a reference quarantine level, where individuals perform their every-day out-of-household activities (i.e. working, shopping, socialising) as normal, for an average 10 hours per day. Medium adherence to community quarantine restricts every-day out-of-household activities to 50% of normal, i.e. 5 hours a day. A complete community quarantine corresponds to no out-of-household activities at all, i.e. 0 hours a day. For any degree of quarantine adherence, we tested for four different average household sizes: (i) larger average households of six persons, (ii) medium average households of three persons, (iii) small average households of two persons, and lastly (iv) single-person households. 

**Figure 1:**
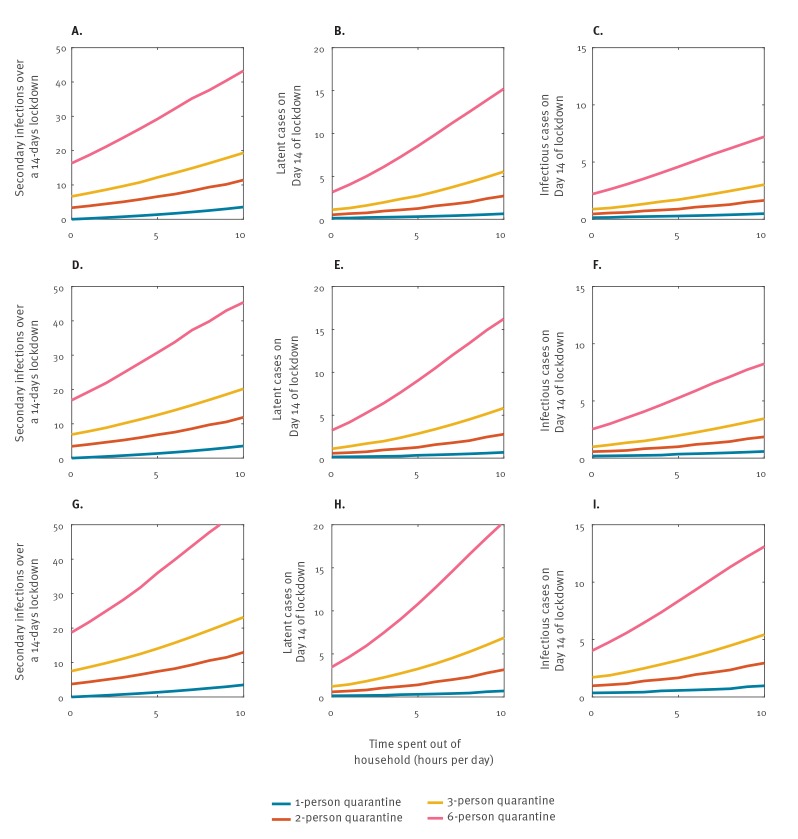
Estimation of the number of total secondary cases (panels A, D, G) during the 14-days period and the (panels B, E, H) latent and (panels C, F, I) infectious cases of COVID-19 at the end of a 14-days lockdown of a city with a population of 5,000 persons, depending on the degree of community quarantine adherence^a^, the size of quarantine units and the proportion of asymptomatic cases

Given that Italy had initially implemented a 14-days lockdown with community quarantine in several towns in Northern Italy, we estimated the number of secondary cases, including asymptomatic cases, in a town of 5,000 persons by the end of this time period in relation to the above scenarios (Figure 1). For any degree of quarantine adherence between 0 and 10 hours, Figure1A, Figure 1B and Figure 1C provide the expected number of secondary infections over 14 days of lockdown, and the number of latent infectious persons and the number of infectious persons respectively at Day 14 of lockdown. These reported numbers relate to a population where 10% of all infected persons are asymptomatic, and would increase for 20% (Figure 1 D–F) or 50% (Figure 1 G–I) asymptomatic cases.

COVID-19: coronavirus disease.

^a^ Low adherence means that a large amount of time (up to 10 hours) is spent out of the household per day and per person.

Generally, higher adherence and smaller quarantine units help to minimise the number of (Panel A) secondary cases over the 14 days period, as well as (Panel B) latent and (Panel C) infectious cases at the end of a lockdown. This was shown for 10% asymptomatic cases. The same conclusion follows when also considering (Panels D–F) 20% asymptomatic cases and (Panels G–I) 50% asymptomatic cases. The number of cases, however, increases with the proportion of asymptomatic cases.

Our model shows how the number of secondary cases within the town increases with the time spent in the public, and also with the average household size (i.e. the quarantine unit). Looking at the extremes, for a six-person household and no community quarantine, we predicted 43 new infections over the 14 days period. In contrast, for a single-person household and complete community quarantine (no time outside of homes), no secondary cases were predicted over the 14 days period. The average household size in Italy is 2.58 according to the Organisation for Economic Co-operation and Development (OECD) [12]. For an average household size of two persons with complete, near-complete, medium and no community quarantine (i.e. 0, 1, 5, and 10 hours respectively in the community), we predict 3, 4, 7 and 11 secondary infections during the lockdown. With an average three-person household size, 7, 8, 12 and 20 secondary infections are predicted, respectively. The average Italian household size 2.58 is thus in-between that of a two-person and three-person household size. With a six-person average household size, 16, 19, 29 and 43 secondary infections would be predicted to occur over the 14-days period, respectively.

In addition, our model indicates that the number of secondary, latent and infected cases has a linear relationship with the population size of a lockdown region, provided same population densities apply between cities. In a locked-down area with 50,000 people, we would expect for an average household size of two persons with complete, near-complete, medium and no community quarantine 30, 40, 70 and 110 secondary infections over the 14-days period, respectively.

## Duration of the quarantine

**Figure 2:**
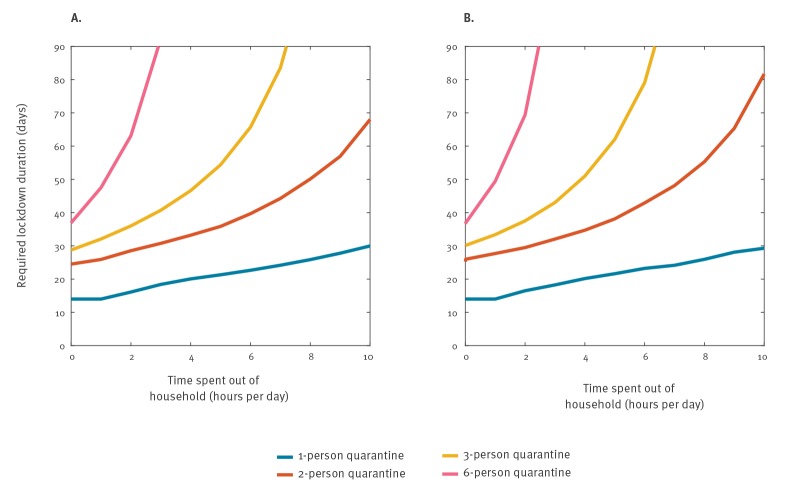
Estimation of the required duration of a lockdown with quarantine, to contain a COVID-19 outbreak in a city of 5,000 inhabitants, depending on the quarantine adherence strictness and household size

The objective of the lockdown with community quarantine is to contain the outbreak within a manageable duration. Figure 2 shows the results on lockdown durations required for average household sizes of 1, 2, 3 and 6, and for various degrees of strictness of quarantine restrictions. Assuming 10% asymptomatic infections (Figure 2a), for a three-person average household-size situation, around 30 days will be a sufficient length under conditions of near-complete community quarantine adherence. With only medium adherence a duration of 54 days would be necessary. Less strict quarantine will result in much longer lockdown periods, which then become unfeasible for any society. These results are only marginally different to a situation with 50% asymptomatic cases (Figure 2b). In addition, the definition to declare an outbreak over requires waiting two times the maximum incubation period after the last case, e.g. 2 × 14 days.

COVID-19: coronavirus disease.

Assuming (panel A) 10% asymptomatic cases, and (panel B) 50% asymptomatic cases respectively, the quarantine duration required to successfully curb the outbreak is presented as a function of time spent out of the household (hours). This is based on that a period of at least 14 days since the last case is a sufficient criterium to suppress the outbreak. Four different quarantine unit group-sizes are shown. The y-axis was limited to 90 days to show the span within the most relevant time-horizon. At this relevant time scale, there are only marginal differences between 10% and 50% asymptomatic cases.

## Discussion

These findings have major implications. A lockdown is designed to reduce spread beyond the lockdown area, and to also prevent further importation into a lockdown area. In a lockdown area, all measures need to be taken to curb transmission. We showed that transmission will continue to occur unless the most stringent community quarantine measures are being taken in a lockdown setting, which means near-complete reduction of all activities in the community. We also find that smaller household sizes, or quarantine group sizes, are associated with fewer secondary cases. Our model results apply to any small-city population size, and can be generalised to larger towns and cities given the linear relationship between population size and secondary cases, assuming a similar population density. COVID-19 is driven by population densities [13]. For settings with higher population densities, which facilitate more intermixing within the population, higher number of secondary cases are expected. The population density in urban Italy is 205.45/km^2^ [14] compared to Hubei Province, China, where it is 2,804/km^2^ [15]. We note that with near 100% restriction of outdoor activities, all transmission will continue to occur within households. In the event of an average household size of three individuals, this would mean that, as a result of within-household transmission, seven secondary cases would be expected (Figure 1a) in a population of 5,000 persons, or 70 secondary infections in a population of 50,000. Public health measures should be in place to immediately test and isolate infected persons.

### Quarantine in the wider context

The lockdown in China with government enforced movement restriction outdoors combined with facility-based case isolation, rigorous contact tracing and quarantine of all contacts, had a substantial impact on interrupting the chain of human-to-human transmission in Wuhan, thus effectively containing the outbreak [16]. While the outbreak in Wuhan involved a highly urbanised setting, the current lockdown in Italy involves small villages with a different social culture and behaviour, and different mechanisms of quarantine enforcement. Our findings suggest that the degree of quarantine adherence needs to be very high regardless of population size in order to be effective. We note, however, that a less strict community quarantine could still flatten the curve of the outbreak compared to no quarantine [17]. In any case, quarantine adherence has an Important and notable impact on reducing the outbreak, but some transmission will still occur within households. We showed that in a theoretical scenario of a single-person household with very strict community quarantine measures, no secondary infections would occur. While a single-person household does not reflect the reality of any society, it does suggest that if all cases could be isolated, e.g. moved out of the community, the epidemic curve would decline much faster and the lockdown duration could be reduced. This means that more efforts need to be done at household level: keeping physical distance even within a household combined with wearing face masks and segregated within-household isolation, or better all symptomatic cases ideally need to be promptly moved out of the household, and isolated in a designated facility. Prompt testing is therefore needed for timely diagnosis and immediate isolation. We also show that a 14-days lockdown period is not sufficient for most scenarios; a longer lockdown duration is needed. On 8 March, Italy announced the need to extend the lockdown to include around 16 million people for 25 additional days until 3 April. Such longer duration should in fact be expected to be required, to have a positive impact, which is going to be very challenging for affected communities to be supplied in food, essential services and to be able to cope psychologically. If lockdown is enforced, it must be done rigorously to truly interrupt transmission, and this would mean near 100% restriction of contacts between persons within the community combined with prompt isolation of new cases. Less strict quarantine adherence would imply even longer lockdown periods, and longer lockdown periods will likely present even greater socioeconomic challenges. By implementing the world’s largest lockdown combined with prompt case isolation, contact tracing of contacts and with strict enforcement of physical distancing [16], containment of COVID-19 in China was shown to be feasible. Remarkably, in South Korea, the control of the outbreak, which had been temporarily lost, was regained without lockdown but with rigorous active case finding, by liberal testing, prompt isolation, and by using novel digital technologies to maximise contact tracing and quarantine of all contacts [18]. In certain places like Taiwan [19], Singapore [20] and Hong Kong, a flat epidemic curve was maintained for COVID-19 by applying very liberal testing, prompt case isolation outside of the community (no home isolation even of the mildest cases), and technologically enhanced contact tracing, very early in the outbreak. If the lockdown in Italy, and meanwhile in many other European countries, is aimed at containment, close to 100% restriction of contact time within communities combined with prompt case detection and immediate isolation of infected persons need to be achieved.

## Supplementary Data

81000 Supplement 1
