# Supplementary material for: Only strict quarantine measures can curb the coronavirus disease (COVID-19) outbreak in Italy, 2020
Source: Euro Surveill. 2020 Apr 2;25(13):2000280. doi: 10.2807/1560-7917.ES.2020.25.13.2000280 (PMC7140595; doi:10.2807/1560-7917.ES.2020.25.13.2000280)
Supplement: Supplement 1 [file 2000280_SJODIN_Supplement1.pdf]

This supplementary material is hosted by *Eurosurveillance* as supporting information alongside the article **Only strict quarantine measures can curb the coronavirus disease (COVID-19) outbreak in Italy, 2020**, on behalf of the authors, who remain responsible for the accuracy and appropriateness of the content. The same standards for ethics, copyright, attributions and permissions as for the article apply. Supplements are not edited by *Eurosurveillance* and the journal is not responsible for the maintenance of any links or email addresses provided therein.

# Supplement 1: Only strict quarantine measures can curb the coronavirus disease (COVID-19) outbreak in Italy, 2020

Henrik Sjödin[1], Annelies Wilder-Smith[2,3,4], Sarah Osman[2], Zia Farooq[1], Joacim Rocklöv[1]

1. Department of Public Health and Clinical Medicine, Umeå University, 901 87 Umeå, Sweden

2. Department of Epidemiology and Global Health, Umeå University, 901 87 Umeå, Sweden;

3. Department of Disease Control, London School of Hygiene and Tropical Medicine, UK;

4. Heidelberg Institute of Global Health, University of Heidelberg, Germany

Correspondence: henrik.sjodin@umu.se

## Master equation

We developed a SEIR epidemiological model in the form of a Master equation (Keeling & Rohani 2008; Gardiner 2009). The model is designed specifically to study small-scale communities under a various degrees of intervention measures in the form of quarantined clusters of people, and where quarantined individuals leave the quarantine from time to time and enter public locations. Any individual upon confirmed infection are isolated, i.e., removed from the small-scale community (i.e., moved to a hospital).

Let $X$, $Y$, $Z$ and $\Omega$ represent random variables corresponding to $S$, $E$, $I$ and $R$, where $[X, Y, Z, \Omega]$ can respectively take any realized values $[(i = 0,1,\dots,n), (j = 0,1,\dots,n), (k = 0,1,\dots,n), (l = 0,1,\dots,n)]$, with the restriction that $i + j + k + l = n$; that is, the sum of individuals in different compartments cannot exceed the household group-size $n$. The household group-size should be perceived as an average group size. However, the model does not explicitly consider the distribution of group sizes, and therefore also not the variation around this mean. The results will be most valid for household-size distributions with smaller variances. Let $p_{ijkl}$denote the probability of finding the system in state $s = (i, j, k, l)$. The Master equation can then be written as:

$$\begin{aligned} \frac{dp_s}{dt} = {} & p_{(i+1,j-1)}\left(\frac{(i+1)k\beta}{n-l}(1-\varphi) + \varphi\left(\frac{\big(i+1-o(i+1,l)\big)k\beta}{n-l-o(i+1,l)} + M(i+1,j-1)\right)\right) \\ & + p_{(j+1,k-1)}\left(\frac{j+1}{L}\right) + p_{(k+,l-)}(k+1)\big((1-a)\gamma + a\gamma_a\big) \\ & - p_s\left(\frac{\beta ik}{(n-l)}(1-\varphi) + \varphi\left(\frac{\big(i-o(i,l)\big)k\beta}{n-l-o(i,l)} + M(i,j)\right) + \frac{j}{L} \right. \\ & \left. + k\big((1-a)\gamma + a\gamma_a\big)\right);\ o(i,l) = \frac{\sigma(n-l)im}{n-l}, \end{aligned} \tag{S1}$$

where the within-cluster per-capita rate of infection $\beta$ is the product between within-cluster contact-rate $c$ and the probability of disease transmission $\tau$. The parameter $\gamma$ denotes the recovery- or removal rate, $\gamma_a$ that for the proportion $a$ of asymptomatic cases, and $L$ the latent period. The parameter $\varphi$ denotes the proportion of a full day is spent by any person outside of the household at common locations, e.g., at work, at schools, in the city center, at shopping malls. The parameter $\varphi$ should be perceived as the average proportion in the population. However, the model does not explicitly consider the distribution of proportions, and therefore also not the variation around the mean $\varphi$. The results will be most valid for proportion distributions with smaller variances. To model the degree of adherence to quarantine regulations, or the lack thereof (e.g., the process of individuals leaving the quarantine temporarily for supplies at a common location), a social mixing process was accounted for in the form:

$$M(i,j,k) = \left(\frac{i}{n-l}\right) m\, \sigma(n-l)\, \tilde{F}; \qquad \tilde{F} = \tilde{\beta}\frac{\tilde{I}}{\tilde{N}},$$

where $\left(\frac{i}{n-l}\right) m\, \sigma(n-l)$ is the rate at which susceptible individuals in a quarantine state $(i,j,k,n-l)$ leave the quarantine, with $m$ being the per-capita rate of leaving the quarantine, and $\sigma(i)$ is a function defining potential state dependent leaving rates, here we set $\sigma(x) = x$. This is multiplied by the common-location force of infection $\tilde{F} = \tilde{\beta}\frac{\tilde{I}}{\tilde{N}}$ which can be derived from the following relationships:

$$\tilde{I} = \varphi m Q \sum_s p_s \frac{k}{i+j+k}\, \sigma(n-l) = \langle \frac{Z}{X+Y+Z} \sigma(X+Y+Z) \rangle\, \varphi m Q$$

$$\tilde{S} = \varphi m Q \sum_s p_s \frac{i}{i+j+k}\sigma(n-l) = \langle \frac{X}{X+Y+Z} \sigma(X+Y+Z) \rangle\, \varphi m Q$$

$$\tilde{N} = \langle \sigma(X+Y+Z) \rangle \varphi m Q,$$

and, hence,

$$\tilde{F} = \tilde{\beta}\frac{\tilde{I}}{\tilde{N}} = \frac{\tilde{c}\tau}{\langle \sigma(X+Y+Z) \rangle} \langle \frac{Z\,\sigma(X+Y+Z)}{X+Y+Z} \rangle,$$

where $Q$ is the number of quarantine clusters, $\tilde{S}$ and $\tilde{I}$ the expected number of common-location susceptible and infectious individuals, $\tilde{N}$ the expected total number of common-location individuals, and $\tilde{c}$ the common-location contact-rate. The force of infection in common locations is given by $\tilde{F}$. Note that the total number of individuals in each quarantine-unit was set to $n-l$, i.e., the quarantine unit group-size minus the removed and isolated number of persons $l$. Accordingly, the model accounts for removal of isolated infected individuals, and for the increased force of infection in quarantine units as the realized group-sizes becomes reduced by the removal of the infectious persons.

The master equation (eq. S1) was numerically solved in Matlab using the built-in ordinary differential-equation solver ode45.

## Model assumptions and parameterization

The model assumed the following: all symptomatic cases would be removed from the locked-down town as soon as symptoms occur and test positive. We took into account an incubation time of five days (Li *et al.* 2020), a pre-symptomatic period of infectiousness of one day, and a symptomatic infectious period of another day (awaiting test results). This means a latent period of four days, and symptomatic persons are infectious in the city population during 2 days. Out of all infected persons, however, 10%, 20% or 50% was assumed to remain asymptomatic (Mizumoto *et al.* 2020; Nishiura *et al.* 2020), and therefore not isolated as they remain undetected. We assumed that this proportion of infected persons had an infectious period of 10 days as suggested by Zou et al. (2020). We modelled a scenario where 0.1% of the population in the high-risk town would be infected (in the latent period) at the time of implementing

the quarantine policy, and all infected symptomatic persons would have been already removed from the town(in hospital isolation).

## The expected time between infected cases

The expected time between new infected cases, i.e., the time since last infected case, was derived by taking the inverse of the average daily rate of infection, which can be written

$$\left(\sum_i \sum_j \sum_k \sum_l p_{ijkl}\left[\frac{\beta ik}{(n-l)}(1-\varphi)+\varphi\left(\frac{\big(i-o(i,l)\big)k\beta}{n-l-o(i,l)}+\ M(i,j)\right)\right]\right)^{-1}.$$

## References


Gardiner, C. (2009). *Stochastic Methods: A Handbook for the Natural and Social Sciences*. 4th ed. 2009 edition. Springer, Berlin.

Keeling, M.J. & Rohani, P. (2008). *Modeling Infectious Diseases in Humans and Animals*. Princeton University Press, Princeton, New Jersey.

Li, Q., Guan, X., Wu, P., Wang, X., Zhou, L., Tong, Y., *et al.* (2020). Early transmission dynamics in wuhan, china, of novel coronavirus–infected pneumonia. *New England Journal of Medicine*.

Mizumoto, K., Kagaya, K., Zarebski, A. & Chowell, G. (2020). Estimating the asymptomatic proportion of coronavirus disease 2019 (COVID-19) cases on board the Diamond Princess cruise ship, Yokohama, Japan, 2020. *Eurosurveillance*, 25, 2000180.

Nishiura, H., Kobayashi, T., Suzuki, A., Jung, S.-M., Hayashi, K., Kinoshita, R., *et al.* (2020). Estimation of the asymptomatic ratio of novel coronavirus infections (COVID-19). *International Journal of Infectious Diseases*, 0.

Zou, L., Ruan, F., Huang, M., Liang, L., Huang, H., Hong, Z., *et al.* (2020). Sars-cov-2 viral load in upper respiratory specimens of infected patients. *New England Journal of Medicine*.
